# Weighing Scale-Based Pulse Transit Time is a Superior Marker of Blood Pressure than Conventional Pulse Arrival Time

**DOI:** 10.1038/srep39273

**Published:** 2016-12-15

**Authors:** Stephanie L.-O. Martin, Andrew M. Carek, Chang-Sei Kim, Hazar Ashouri, Omer T. Inan, Jin-Oh Hahn, Ramakrishna Mukkamala

**Affiliations:** 1 Department of Mechanical Engineering, University of Maryland, College Park, MD, USA; 2 School of Electrical and Computer Engineering, Georgia Institute of Technology, Atlanta, GA, USA; 3 Department of Electrical and Computer Engineering, Michigan State University, East Lansing, MI, USA

## Abstract

Pulse transit time (PTT) is being widely pursued for cuff-less blood pressure (BP) monitoring. Most efforts have employed the time delay between ECG and finger photoplethysmography (PPG) waveforms as a convenient surrogate of PTT. However, these conventional pulse arrival time (PAT) measurements include the pre-ejection period (PEP) and the time delay through small, muscular arteries and may thus be an unreliable marker of BP. We assessed a bathroom weighing scale-like system for convenient measurement of ballistocardiography and foot PPG waveforms – and thus PTT through larger, more elastic arteries – in terms of its ability to improve tracking of BP in individual subjects. We measured “scale PTT”, conventional PAT, and cuff BP in humans during interventions that increased BP but changed PEP and smooth muscle contraction differently. Scale PTT tracked the diastolic BP changes well, with correlation coefficient of −0.80 ± 0.02 (mean ± SE) and root-mean-squared-error of 7.6 ± 0.5 mmHg after a best-case calibration. Conventional PAT was significantly inferior in tracking these changes, with correlation coefficient of −0.60 ± 0.04 and root-mean-squared-error of 14.6 ± 1.5 mmHg (p < 0.05). Scale PTT also tracked the systolic BP changes better than conventional PAT but not to an acceptable level. With further development, scale PTT may permit reliable, convenient measurement of BP.

Pulse transit time (PTT) is the time elapsed for the pressure wave to travel between two arterial sites. According to the well-known Moens-Kortweg equation, PTT decreases as the arteries stiffen. Since arterial stiffness increases with blood pressure (BP) via the mechanical properties of the arterial wall, PTT often shows a tight, inverse relationship with BP in individual subjects. Moreover, PTT can be measured simply as the relative timing between proximal and distal waveforms indicative of arterial pulsations. For these reasons, PTT is being widely investigated at present to achieve convenient, cuff-less BP monitoring (see recent reviews^1^,^2^,^3^).

Most previous studies of BP measurement via PTT have used the time delay between an electrocardiography (ECG) waveform and an arterial waveform from an arm, especially a finger blood volume waveform via a photoplethysmography (PPG) sensor, as a convenient surrogate of PTT^1^. However, these “conventional pulse arrival time (PAT)” measurements have two shortcomings. The first shortcoming is that PAT includes the pre-ejection period (PEP) in addition to PTT. Since the PEP component depends on the electromechanical functioning of the heart, it can change independently of PTT and thus BP. For example, PEP changes in the same direction as PTT during exercise^4^ but in the opposite direction of PTT during vasoconstriction^5^. Note that several studies of PAT have demonstrated good correlation with BP by employing only exercise-induced BP changes^1^. The second shortcoming is that the PTT component represents the time delay through mainly small arteries. In such arteries, smooth muscle contraction and relaxation can cause variations in arterial stiffness, and thus PTT, that are independent of BP. For example, arm PTT increases despite no change in diastolic BP during vasodilation^6^.

In a recent study, we developed a system in a form similar to a bathroom weighing scale for convenient measurement of PTT through larger, more elastic arteries including the aorta. The “weighing scale” comprises a ballistocardiography (BCG) sensor to measure a proximal waveform indicative of the mechanical timing of aortic ejection^7^,^8^ when the subject stands on it and a PPG sensor to simultaneously acquire a distal blood volume waveform from the foot.

In this study, we compared PTT measurements from the scale with conventional PAT measurements as markers of BP in human volunteers subjected to multiple interventions that changed BP via different physiologic mechanisms. Our results showed that the weighing scale-based PTT measurements tracked the BP changes in individual subjects significantly better than the conventional measurements without much compromise in convenience.

## Methods

### Data Collection

We studied human subjects under a protocol approved by the Georgia Institute of Technology and Michigan State University Institutional Review Boards (IRBs) and performed procedures in accordance with the guidelines and regulations of both IRBs. We recruited 22 adults for study and obtained written, informed consent. The volunteers were young and healthy (age: 25 ± 3.5 years; gender: 19 males; height: 177 ± 11 cm; weight: 75 ± 15 kg).

**Figure 1:**
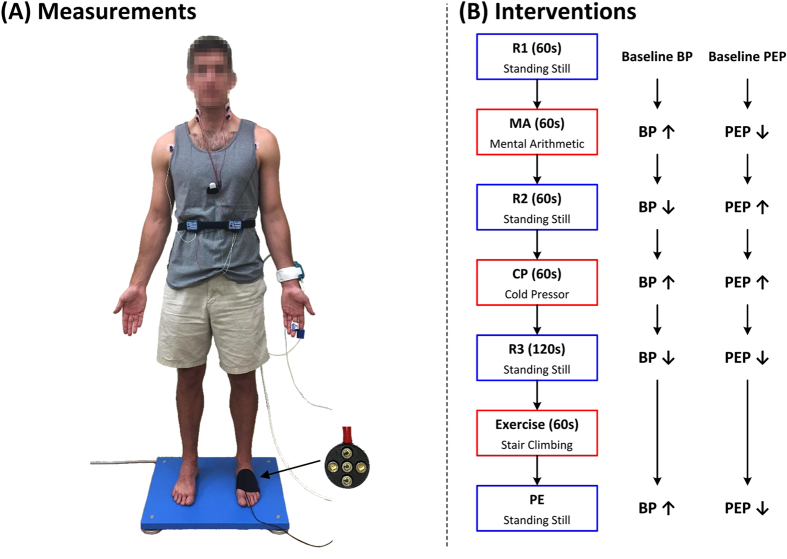
Data collection for comparing weighing scale-based pulse transit time (PTT) to conventional pulse arrival time (PAT) as markers of blood pressure (BP) in humans. (**A**) Ballistocardiography (BCG) and foot photoplethysmography (PPG) waveforms were measured with a custom system similar to a weighing scale; ECG, finger PPG, and impedance cardiography (ICG) waveforms were measured with standard sensors; and a reference finger cuff BP waveform was measured with a volume-clamp device. (**B**) The waveforms were recorded during three baseline periods (R1, R2, R3) and three interventions (mental arithmetic (MA), cold pressor (CP), post-exercise (PE)) to increase BP but change the pre-ejection period (PEP) differently.

For each subject, we measured six physiologic waveforms, as shown in Fig. 1A. We placed three gel electrodes on the chest in Lead II configuration and interfaced the electrodes to a wireless amplifier (BN-EL50, Biopac Systems, Goleta, CA, USA) for an ECG waveform; eight gel electrodes on the neck and thorax in standard impedance cardiography (ICG) configuration and interfaced the electrodes to a wireless electrical bioimpedance module (BN-NICO, Biopac Systems) for an impedance waveform differentiated with respect to time (“ICG waveform”); a transmission-mode PPG clip (8000AA, Nonin Medical, MN, USA) on an index finger and attached the sensor to a wired PPG module (PPG100C, Biopac Systems) for a finger blood volume waveform (“finger PPG waveform”); and a finger cuff embedded with a PPG sensor on the middle finger of the same hand to implement the volume-clamp method (ccNexfin, Edwards Lifesciences, Irvine, CA, USA) for a reference cuff BP waveform. We then asked the volunteer to stand on the custom weighing scale-like system that we previously developed. This system consists of a high resolution force plate (Type 9260AA6, Kistler Group, Winterthur, Switzerland) for a BCG waveform^9^ and a PPG sensor array within an adjustable strap for a blood volume waveform from the instep of the foot (“foot PPG waveform”). We interfaced all of the measurement devices to a laptop computer via a data acquisition unit (MP150, Biopac Systems) and recorded the waveforms at a 2 kHz sampling rate. Note that we obtained the ICG waveform, which is a conventional, but relatively inconvenient, non-invasive hemodynamic measurement indicative of the proximal arterial timing, as an additional benchmark for comparison.

We collected the data during three hemodynamic interventions, as shown in Fig. 1B. First, after standing still for 60 sec to obtain an initial baseline recording (R1), the subject repeatedly added digits of a three-digit number and then added the sum to the original number for 60 sec to obtain a mental arithmetic recording (MA)^10^. Second, the subject rested for 60 sec to obtain another baseline recording (R2) and then immersed her/his free hand into 4 °C water for 60 sec to obtain a cold pressor recording (CP)^11^. Finally, after resting for 120 sec to obtain a third baseline recording (R3), the subject got off the scale, performed a stair-climbing exercise for 60 seconds, and then returned on the scale to obtain a post-exercise recording (PE)^12^. We designed the study to include these interventions, because they are safe, induce little motion artifact, mimic activities that occur in daily life (e.g., public speaking, experiencing cold weather, taking a seat after walking up a flight of stairs), and are known to increase BP but change PEP differently^13^,^14^,^15^,^16^,^17^. For example, MA and PE decrease PEP due to enhanced ventricular contractility, whereas CP increases PEP due to enhanced afterload.

### Data Analysis

For each subject record, we selected segments from the baseline periods (R1, R2, R3) and interventions (MA, CP, PE). This process yielded six sets of six waveform segments per subject for the 22 subjects.

**Figure 2:**
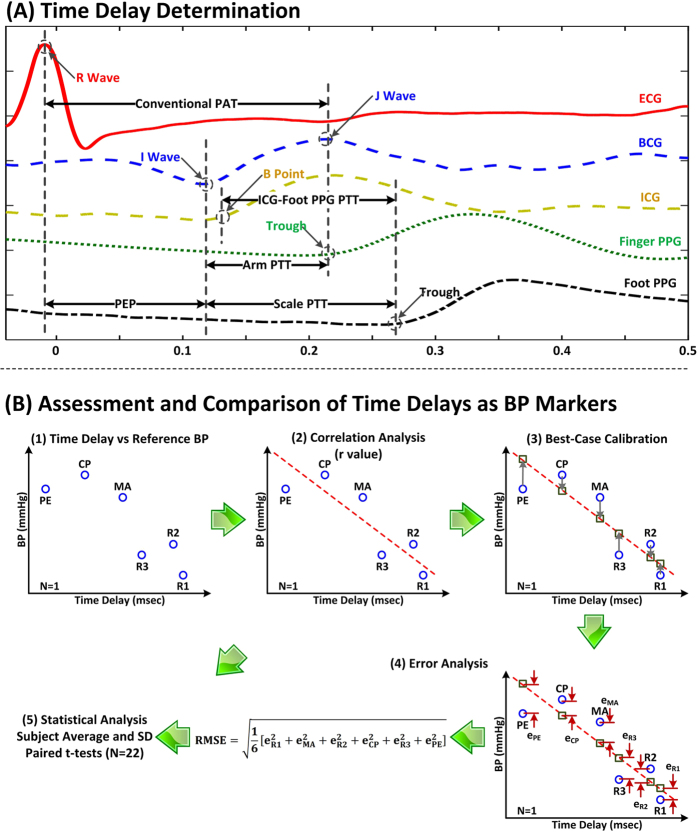
Data analysis for comparing scale PTT to conventional PAT as markers of BP in humans. (**A**) Scale PTT, conventional PAT, PEP, and other time delays were detected from the waveforms. (**B**) The time delays were assessed and compared in terms of their ability to track the intervention-induced BP changes via the correlation coefficient (r) and root-mean-squared-error (RMSE) after a best-case calibration.

As shown in Fig. 2A, for each set of waveform segments, we determined (i) weighing scale-based PTT (scale PTT for short) as the time delay between the BCG I wave, which may denote the timing of pulsation specifically in the aortic arch^7^, and the foot PPG trough; (ii) conventional PAT as the time delay between the ECG R wave and finger PPG trough; (iii) PEP as the time delay between the ECG R wave and BCG I wave; (iv) “arm PTT” as the time delay between the BCG I wave and finger PPG trough; and (v) “ICG-foot PPG PTT” as the time delay between the ICG B-point and foot PPG trough. We used the troughs of the PPG waveforms as opposed to other possible fiducial points based on our earlier findings^18^. We automatically determined the five time delays as follows.

We first band-pass filtered the BCG, PPG, and ICG waveform segments using a first-order Butterworth filter with cutoff frequencies of 0.5 and 10 Hz. We further smoothed the BCG waveform segments using an exponential filter to suppress motion artifact^19^. We then detected the fiducial points of interest for each beat as follows. We detected the ECG R wave using the popular Pan-Tompkins method. To detect the BCG I wave, we first identified the more prominent J wave as the maximum within 100 ms to 300 ms after the ECG R wave. We then identified the I wave as the local minimum between the ECG R wave and J wave nearest to the J wave. We detected the PPG troughs by applying the intersecting tangent method between successive ECG R waves^18^,^19^,^20^ and the ICG B point as the maximum of the first derivative between consecutive R waves (which turned out to be the best amongst various possible fiducial points). We next took the differences between the times of appropriate pairs of fiducial points to determine the time delays for each beat. We also detected the minimum and maximum of the cuff BP waveform between successive ECG R waves to establish reference diastolic and systolic BP for each beat. We finally averaged the time delays and reference BP levels, excluding outliers, over five beat intervals and selected the interval wherein average diastolic BP was minimal for each baseline period and maximal for each intervention to attain maximal BP effect.

We assessed and compared the time delays as markers of BP, as shown in Fig. 2B. We first computed the correlation coefficient (r) between a time delay and each reference BP level per subject. To present the data from all of the subjects at once, we also calibrated a time delay to each reference BP level by finding the line that best fitted the pairs of the time delays and BP levels for a subject and then mapping the time delays of that subject through the line so as to predict the BP levels. We then computed the root-mean-square-error (RMSE) between the predicted and reference BP levels per subject as an indication of the best-case BP measurement accuracy offered by the time delay. We finally compared the time delays via paired t-tests of the correlation coefficients and RMSEs.

## Results

**Figure 3:**
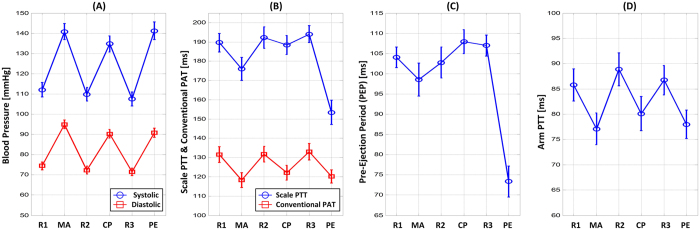
Group average (mean ± SE for N = 22) of (**A**) diastolic and systolic BP, (**B**) scale PTT and conventional PAT, (**C**) PEP, and (**D**) arm PTT for each baseline period and intervention. In contrast to conventional PAT, scale PTT was able to correctly track the BP changes on average mainly due to the elimination of PEP but perhaps also due to mitigation of the impact of smooth muscle contraction following the CP intervention.

Figure 3A illustrates the group average (mean ± SE) cuff diastolic and systolic BP for the three baseline periods (R1, R2, R3) and three interventions (MA, CP, PE). Each intervention caused the BP levels to increase by a significant and similar extent (about 20 mmHg for diastolic BP and 30 mmHg for systolic BP). Figure 3B shows the corresponding group average scale PTT and conventional PAT. Both time delays correctly changed in the opposite direction of the BP changes. Importantly, however, the magnitudes of the changes in the two time delays were different. Scale PTT correctly decreased by a similar amount in response to each intervention, whereas conventional PAT appeared to under-respond to CP and over-respond to PE. Figure 3C shows the group average PEP. PEP was clearly responsible for the inconsistent changes in conventional PAT relative to BP. PEP decreased in response to MA but increased in response to CP and decreased considerably in response to PE. Figure. 3D shows the group average arm PTT. PEP was not singularly responsible for the inconsistent changes in conventional PAT relative to BP, as the arm PTT component did not increase enough from CP to R3. CP may cause smooth muscle contraction^11^, which would further reduce arm PTT. Smooth muscle contraction could therefore explain why arm PTT under-responded to R3. On the other hand, arm PTT did not over-respond to CP, which is against the smooth muscle contraction hypothesis.

**Figure 4:**
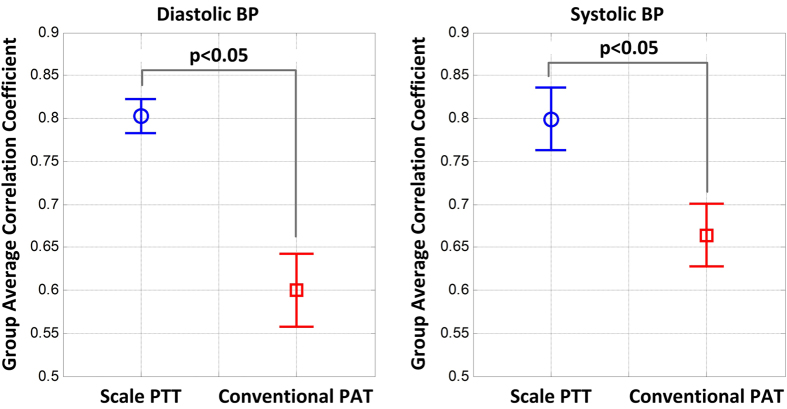
Group average correlation coefficients between scale PTT and each BP level and conventional PAT and each BP level. Scale PTT correlated with both diastolic and systolic BP significantly better than conventional PAT.

**Figure 5:**
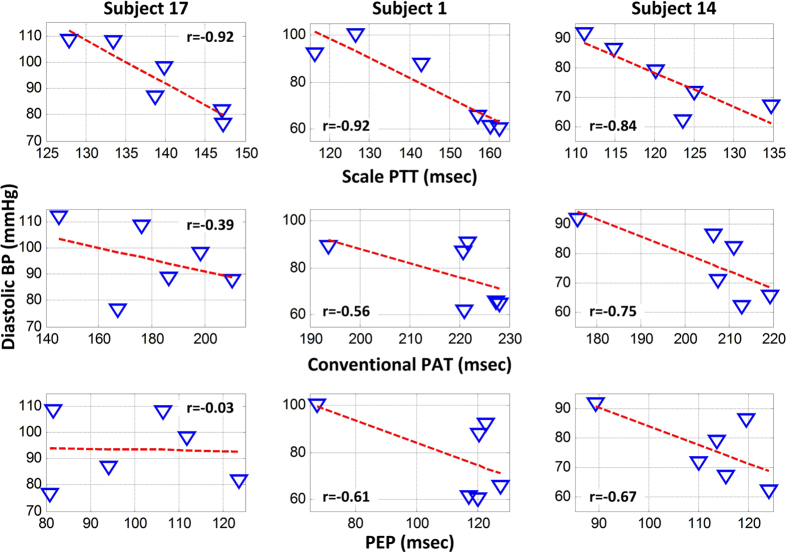
Representative correlation plots of diastolic BP versus scale PTT and versus conventional PAT in three subjects. The improved BP correlation offered by scale PTT varied from subject to subject mainly due to the variable performance of conventional PAT and its PEP and arm PTT components.

Figure 4 shows the group average correlation coefficients (r) between scale PTT and each of cuff diastolic and systolic BP and between conventional PAT and each cuff BP level, while Fig. 5 illustrates representative examples of the correlation plots of these time delays versus cuff diastolic BP and of PEP versus cuff diastolic BP in three of the subjects. In accordance with Fig. 3, scale PTT correlated with both BP levels fairly well (r = −0.80) and better than conventional PAT. On average, scale PTT showed 33 and 21% higher correlations with diastolic BP and systolic BP, respectively. This improvement over conventional PAT was statistically significant. However, there was nontrivial variability in the improvement from subject to subject mainly due to the variable performance of conventional PAT. This time delay did not correlate well with the BP levels in about a third of the subjects due to the PEP component (see example in left panels of Fig. 5) and in about another third of the subjects due to the arm PTT component (middle panels) but did correlate well with the BP levels in the remaining subjects (right panels).

**Figure 6:**
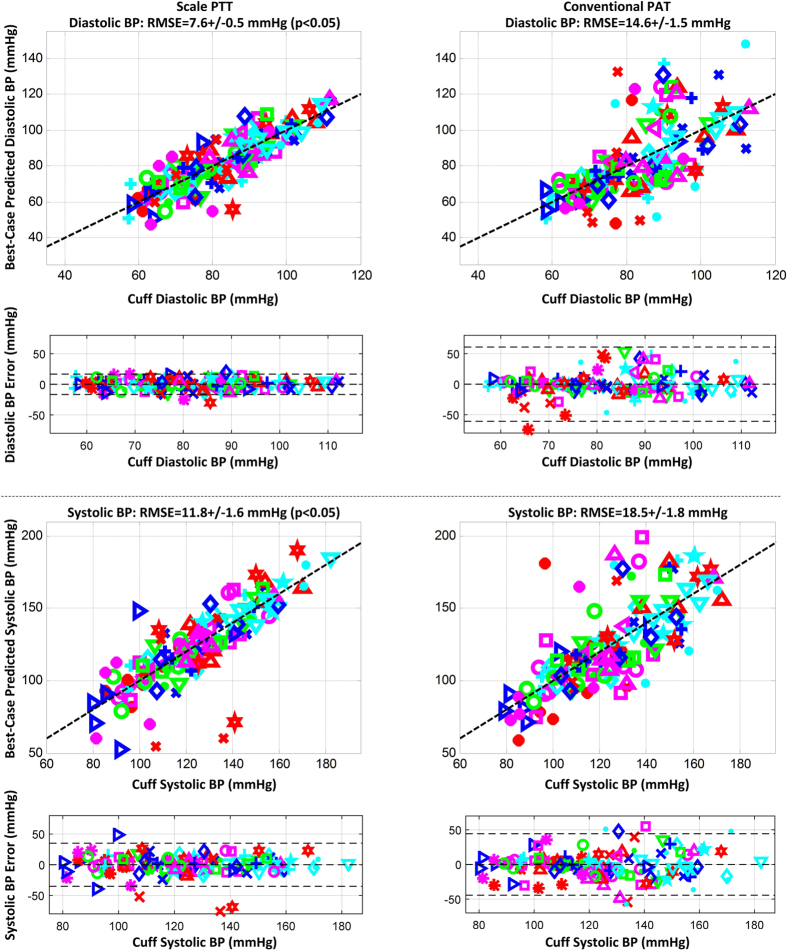
Correlation plots of predicted BP via scale PTT and via conventional PAT after best-case calibration for each time delay versus cuff BP and Bland-Altman plots of the errors between the predicted and measured BP versus cuff BP pooled over all the subjects, along with the group average RMSEs. The different symbols correspond to each of the subjects. Scale PTT yielded a good diastolic BP RMSE, whereas conventional PAT produced unacceptable diastolic and systolic BP RMSEs.

Figure 6 illustrates correlation plots of the best-case calibrated BP levels predicted by scale PTT and conventional PAT versus the cuff BP levels and Bland-Altman plots of the errors between the predicted and measured BP levels versus the cuff BP levels over all of the subjects. The figure also shows the group average RMSEs (after removal of one outlier subject for each time delay). Scale PTT yielded a good diastolic BP RMSE (7.6 ± 0.5 mmHg) that was 48% lower than that of conventional PAT. Scale PTT also provided a systolic BP RMSE that was 36% lower than that of conventional PAT. However, despite also being best-case, the systolic BP RMSE of scale PTT was still not good (11.8 ± 1.6 mmHg). Scale PTT was thus able to track the intervention-induced changes in diastolic BP better than systolic BP. Note that the correlation coefficients between scale PTT and each BP level were similar, as a larger data range (i.e. greater systolic than diastolic BP changes here) is known to inflate this metric^21^.

Finally, scale PTT also tended to track the BP changes better than arm PTT in accordance with Fig. 3 and followed the BP changes to a similar degree as less convenient ICG-foot PPG PTT. For example, arm PTT and ICG-foot PPG PTT yielded group average correlation coefficients of −0.71 ± 0.04 and −0.79 ± 0.05 with diastolic BP, respectively. In addition, the group average PEP via the time delay between the ECG R wave and ICG B point was similar to that in Fig. 3C for each baseline period and intervention (results not shown).

## Discussion

We compared scale PTT – the time delay between BCG and foot PPG waveforms obtained with a system that could be readily implemented in the form of a bathroom weighing scale (see Fig. 1A) – to conventional PAT – the time delay between ECG and finger PPG waveforms – as markers of BP. We specifically measured these and other time delays and cuff BP during a set of interventions that significantly increased BP, while changing PEP as well as smooth muscle contraction in different ways, in healthy volunteers (see Fig. 1B) and then quantitatively assessed and compared the time delays in terms of their ability to track the intervention-induced BP changes in individual subjects (see Fig. 2B). Scale PTT, which is extracted at the diastolic level of the waveforms (see Fig. 2A), tracked the diastolic BP changes fairly well, with a correlation coefficient of −0.80 ± 0.02 (see Figs 4 and 5) and a best-case RMSE of 7.6 ± 0.5 mmHg after calibration with the reference cuff BP (see Fig. 6). The corresponding quantitative metrics offered by conventional PAT were −0.60 ± 0.04 and 14.6 ± 1.5 mmHg (see Figs 4, 5, 6). The elimination of PEP and perhaps the mitigation of the impact of smooth muscle contraction in scale PTT led to these 30–50% improvements (see Fig. 3). Scale PTT also afforded superior tracking of the systolic BP changes compared to conventional PAT (see Figs 4 and 6). However, even with the best possible calibration, scale PTT could only yield a systolic BP RMSE of 11.8 ± 1.6 mmHg. In sum, scale PTT provided good tracking of diastolic BP changes, whereas conventional PAT did not track the changes in either diastolic or systolic BP with a level of accuracy that is close to acceptable.

Despite wide recognition of its shortcomings, conventional PAT has been employed in most studies of cuff-less BP monitoring based on the PTT principle due to the convenience of its measurement. However, the convenience of the scale PTT measurement may be comparable to standard form factors for conventional PAT measurement involving gel electrodes and a finger PPG clip (see Fig. 1A). While conventional PAT could potentially be obtained with more convenient form factors (e.g. a wristwatch requiring the placement of the opposite hand on the watch), the substantial improvement in tracking BP changes offered by scale PTT may be worth any convenience trade-off. Note that, as also found here, scale PTT can track BP changes similarly to PTT derived with ICG – a conventional hemodynamic measurement modality that likewise provides a measure of the true proximal arterial timing – while offering an appreciable upgrade in convenience (see Fig. 1A).

In previous studies, we also demonstrated the advantage of PTT over PAT as a marker of BP. In particular, we showed that invasive PTT was significantly better than invasive PAT in terms of tracking large BP changes induced by diverse hemodynamic interventions in animals^5^. However, the use of invasive measurements and animals were limitations. We then showed that non-invasive PTT via a pair of PPG waveforms was superior to non-invasive PAT in terms of BP tracking in a similar animal model^18^. However, the use of animals remained a shortcoming. We also showed that PTT via BCG and finger cuff BP waveforms correlated better with diastolic BP than PAT via ECG and finger cuff BP waveforms in healthy humans during interventions that changed BP but also induced confounding motion artifact^19^. While the use of human subjects was relevant, the distal waveform represented a limitation in that it was not realistic of a cuff-less BP monitoring application and did not permit PTT measurement through larger, more elastic arteries. The present study overcomes these major limitations of our earlier efforts. Moreover, compared to our previous human study, the present study shows the efficacy of BCG-based PTT more clearly.

This study, however, still has limitations. The major limitation is that only young, healthy subjects were studied. On the other hand, younger subjects do represent a sub-population that is particularly important in the sense of early detection of hypertension. Another limitation is that, for convenience, the ECG waveform was used to detect the fiducial points of the BCG and foot PPG waveforms. However, derivation of scale PTT without ECG gating is a feasible engineering task that would surely leverage foot PPG gating to detect the BCG I wave.

Future efforts are needed to bring the weighing scale-like system to practice. First, additional sensing and/or signal processing are needed for integration within the scale to achieve reliable tracking of systolic BP. As outlined in Kim *et al*.^7^, BCG waveform features could possibly be used for this purpose. Second, a calibration procedure is needed to convert scale PTT in units of msec to BP in units of mmHg. One possible, though admittedly not that convenient, procedure is for a subject to stand on the scale with an automatic arm cuff during simple interventions to perturb BP such as those employed herein. A line could then be fitted to the multiple pairs of scale PTT and BP measurements. This line could thereafter be used as a subject-specific calibration curve to achieve cuff-less BP monitoring using the scale alone. Note that the calibration curve would have to be periodically updated (e.g., annually) to account for slow arterial stiffness changes with aging and disease. Finally, as alluded to above, testing of the scale in diverse subjects including the elderly and hypertensive patients are needed to prove broad applicability of the system.

With such successful future efforts, the system – as implemented in the form of an actual bathroom weighing scale – could be used for convenient, cuff-less BP monitoring at home to facilitate hypertension control in an individual patient as well as in a gym locker room to screen for hypertension in many people. In addition, un-calibrated scale PTT could potentially be applied for monitoring large artery stiffness. In particular, carotid-femoral PTT, in the form of pulse wave velocity, is a proven independent cardiovascular risk factor^22^. Yet, because carotid-femoral pulse wave velocity requires tonometer or ultrasound measurements from the neck and groin, it has not become a routine clinical measurement. The convenient scale could thus allow large artery stiffness monitoring to make it to clinical practice.

## Additional Information

**How to cite this article:** Martin, S. L.-O. *et al*. Weighing Scale-Based Pulse Transit Time is a Superior Marker of Blood Pressure than Conventional Pulse Arrival Time. *Sci. Rep.* **6**, 39273; doi: 10.1038/srep39273 (2016).

**Publisher’s note:** Springer Nature remains neutral with regard to jurisdictional claims in published maps and institutional affiliations.
