# Author Correction: Weighing Scale-Based Pulse Transit Time is a Superior Marker of Blood Pressure than Conventional Pulse Arrival Time

**DOI:** 10.1038/s41598-018-34167-3

**Published:** 2018-10-29

**Authors:** Stephanie L.-O. Martin, Andrew M. Carek, Chang-Sei Kim, Hazar Ashouri, Omer T. Inan, Jin-Oh Hahn, Ramakrishna Mukkamala

**Affiliations:** 1 Department of Mechanical Engineering, University of Maryland, College Park, MD USA; 2 School of Electrical and Computer Engineering, Georgia Institute of Technology, Atlanta, GA USA; 3 Department of Electrical and Computer Engineering, Michigan State University, East Lansing, MI USA

Correction to: *Scientific Reports* 10.1038/srep39273, published online 15 December 2016

This Article contains errors.

Figure 6 is incorrect due to an error when calculating linear regression, where x was inadvertently set as the dependent variable and y as the independent variable.

This error does not cause a change to the main conclusion of this Article, but does result in some changes to the detailed interpretation of the results.

The correct Figure 6 and its accompanying legend appear below as Figure 1.

**Figure 1:**
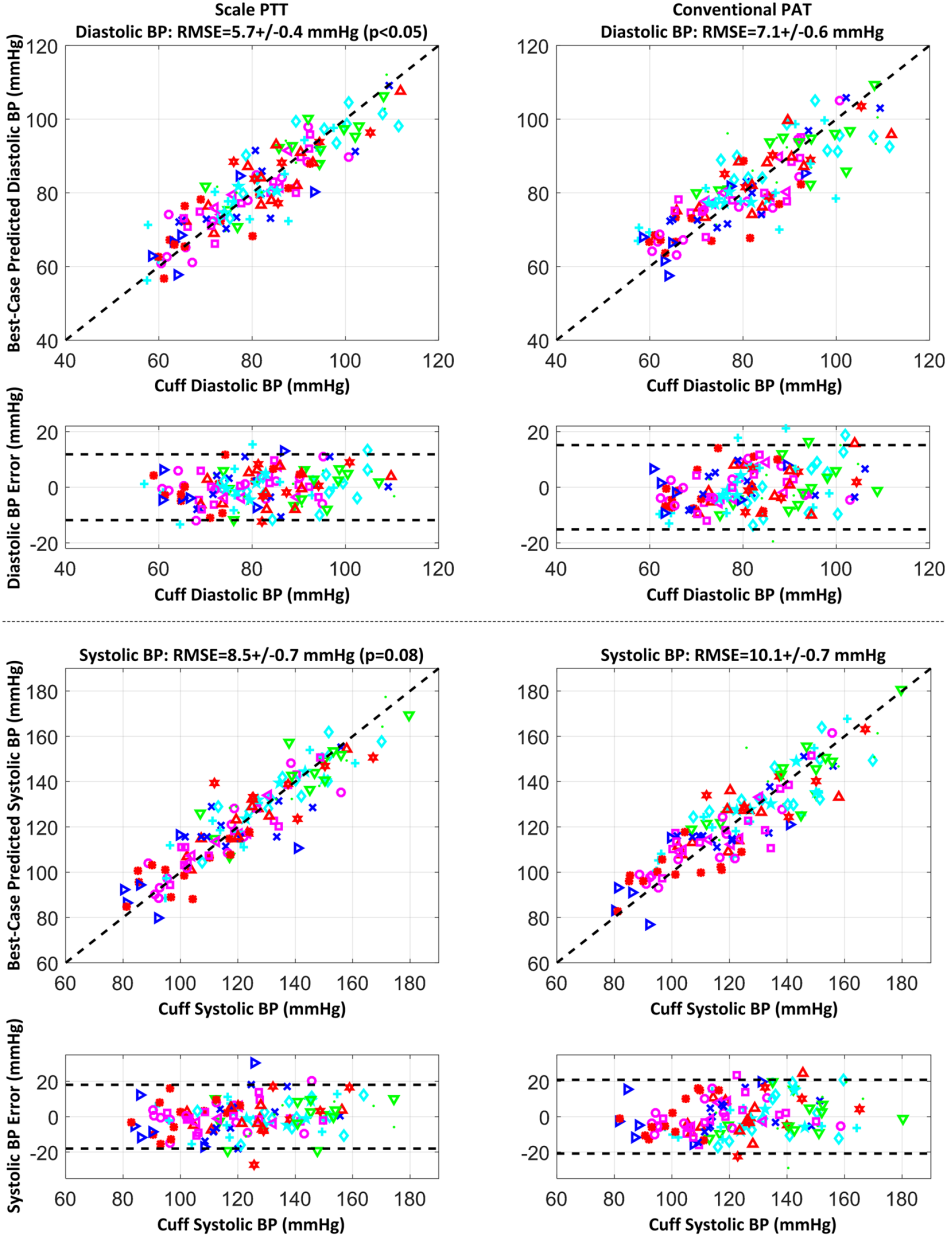
Correlation plots of predicted BP via scale PTT and via conventional PAT after best-case calibration for each time delay versus cuff BP and Bland-Altman plots of the errors between the predicted and measured BP versus cuff BP pooled over all the subjects, along with the group average RMSEs. The different symbols correspond to each of the subjects. Scale PTT yielded lower diastolic and systolic BP RMSEs than conventional PAT..

As a result, in the Abstract,

“Scale PTT tracked the diastolic BP changes well, with correlation coefficient of −0.80 ± 0.02 (mean ± SE) and root-mean-squared-error of 7.6 ± 0.5 mmHg after a best-case calibration. Conventional PAT was significantly inferior in tracking these changes, with correlation coefficient of −0.60 ± 0.04 and root-mean-squared-error of 14.6 ± 1.5 mmHg (p < 0.05). Scale PTT also tracked the systolic BP changes better than conventional PAT but not to an acceptable level.”

should read:

“Scale PTT tracked the diastolic and systolic BP changes well, with correlation coefficients of −0.80 ± 0.02 (mean ± SE) and −0.80 ± 0.04 and root-mean-squared-errors of 5.7 ± 0.4 and 8.5 ± 0.7 mmHg after best-case calibrations. Conventional PAT was significantly inferior in tracking these changes, with corresponding correlation coefficients of −0.60 ± 0.04 and −0.66 ± 0.03 and root-mean-squared-errors of 7.1 ± 0.6 and 10.1 ± 0.7 mmHg (p < 0.05 or 0.10).”

In the Results section,

“Scale PTT yielded a good diastolic BP RMSE (7.6 ± 0.5 mmHg) that was 48% lower than that of conventional PAT. Scale PTT also provided a systolic BP RMSE that was 36% lower than that of conventional PAT. However, despite also being best-case, the systolic BP RMSE of scale PTT was still not good (11.8 ± 1.6 mmHg).”

should read:

“Scale PTT yielded a good diastolic BP RMSE (5.7 ± 0.4 mmHg) that was 20% lower than that of conventional PAT. Scale PTT also provided a good systolic BP RMSE (8.5 ± 0.7 mmHg) that was 15% lower than that of conventional PAT.”

In the Discussion section,

“Scale PTT, which is extracted at the diastolic level of the waveforms (see Fig. 2A), tracked the diastolic BP changes fairly well, with a correlation coefficient of −0.80 ± 0.02 (see Figs 4 and 5) and a best-case RMSE of 7.6 ± 0.5 mmHg after calibration with the reference cuff BP (see Fig. 6). The corresponding quantitative metrics offered by conventional PAT were −0.60 ± 0.04 and 14.6 ± 1.5 mmHg (see Figs 4, 5, 6). The elimination of PEP and perhaps the mitigation of the impact of smooth muscle contraction in scale PTT led to these 30–50% improvements (see Fig. 3). Scale PTT also afforded superior tracking of the systolic BP changes compared to conventional PAT (see Figs 4 and 6). However, even with the best possible calibration, scale PTT could only yield a systolic BP RMSE of 11.8 ± 1.6 mmHg. In sum, scale PTT provided good tracking of diastolic BP changes, whereas conventional PAT did not track the changes in either diastolic or systolic BP with a level of accuracy that is close to acceptable.”

should read:

“Scale PTT, which is extracted at the diastolic level of the waveforms (see Fig. 2A), tracked the diastolic BP changes well, with a correlation coefficient of −0.80 ± 0.02 (see Figs 4 and 5) and a best-case RMSE of 5.7 ± 0.4 mmHg after calibration with the reference cuff BP (see Fig. 6). The corresponding quantitative metrics offered by conventional PAT were −0.60 ± 0.04 and 7.1 ± 0.6 mmHg (see Figs 4, 5, 6). The elimination of PEP and perhaps the mitigation of the impact of smooth muscle contraction in scale PTT led to these 20–33% improvements (see Fig. 3). Because the systolic BP changes paralleled the diastolic BP changes in this study (see Fig. 3), scale PTT was also able to track the systolic BP changes fairly well and better than PAT by 15–20% (see Figs 4 and 6). The diastolic and systolic BP RMSE reduction of 1.5 mmHg afforded by scale PTT may appear modest. However, based on the definition of the correlation coefficient and the fact that the bias error is near zero for best-case calibration, the RMSE here scales with the magnitude of the BP change (i.e., *RMSE = 1-r2σBP*, where *r* is the correlation coefficient and *σBP* is the standard deviation of BP). In this study, the standard deviation of BP due to the interventions was not large (e.g., about 10 mmHg for diastolic BP, as shown in Fig. 3). Hence, the RMSE reduction afforded by scale PTT may become substantial with a more significant BP range. In sum, scale PTT tracked the BP changes with a level of accuracy that was good overall and better than conventional PAT.”

